# Direct Prototyping of Patterned Nanoporous Carbon: A Route from Materials to On-chip Devices

**DOI:** 10.1038/srep02294

**Published:** 2013-07-26

**Authors:** Caiwei Shen, Xiaohong Wang, Wenfeng Zhang, Feiyu Kang

**Affiliations:** 1 Tsinghua National Laboratory for Information Science and Technology, PR China; 2 Institute of Microelectronics, Tsinghua University, Beijing 100084, PR China; 3 Department of Materials Science and Engineering, Tsinghua University, Beijing 100084, PR China

## Abstract

Prototyping of nanoporous carbon membranes with three-dimensional microscale patterns is significant for integration of such multifunctional materials into various miniaturized systems. Incorporating nano material synthesis into microelectronics technology, we present a novel approach to direct prototyping of carbon membranes with highly nanoporous structures inside. Membranes with significant thicknesses (1 ~ 40 μm) are rapidly prototyped at wafer level by combining nano templating method with readily available microfabrication techniques, which include photolithography, high-temperature annealing and etching. In particular, the high-surface-area membranes are specified as three-dimensional electrodes for micro supercapacitors and show high performance compared to reported ones. Improvements in scalability, compatibility and cost make the general strategy promising for batch fabrication of operational on-chip devices or full integration of three-dimensional nanoporous membranes with existing micro systems.

Nanoporous polymer and carbon materials have been intensively studied for diverse scientific and technological applications such as molecular separation, adsorption, sensing, catalysts supporting and so on, owing to their nano-structured thoroughfares, high surface areas and chemical stability^1^,^2^,^3^,^4^,^5^,^6^,^7^,^8^,^9^,^10^. And the naturally conductive porous carbons find additional applications as electrodes in fuel cells, batteries and supercapacitors for energy conversion and storage^11^,^12^,^13^,^14^,^15^,^16^. Polymers or carbons with various pore structures and their pore sizes ranging from subnanometers to submicrometers are accessible through a number of methods, such as chemical activation, self-assembly using soft templates and replication of presynthesized hard templates^17^,^18^. Since the earliest work by Knox and coworkers^19^, the synthetic method involving inorganic hard templates has proven to be one of the most applied and successful approaches, because the resulting porous materials have advantages including well-defined pore structures, tunable pore sizes and narrow pore-size distributions^17^,^18^.

While significant progress has been made on designing nanostructures of porous materials, most of the nanoporous polymers and carbons are synthesized to be irregular blocks or powders. However, prototyping of such nanostructured functional materials into defined patterns will be more attractive for their promising potential applications in different scales. In particular, applications of nanoporous carbons such as energy conversion or storage in miniaturized systems^11^,^20^ require the material to be patterned into three-dimensional (3D) micro structures to provide internal surface areas as high as possible. J. Chmiola et al.^11^ once proposed carbide derived carbon as the functional material for on-chip micro supercapacitors. Such nanoporous carbon materials fabricated by micro fabrication protocols have shown quite large specific capacitance and thus have great potential for on-chip energy storage. But the proposed materials are not easy to be patterned into micro electrodes, and are difficult to be applied into micro devices.

On the other hand, a number of techniques have been proposed to pattern different porous oxides, including soft lithography, micro-pen lithography, ink-jet printing, dipcoating and electron beam lithography^21^,^22^,^23^,^24^,^25^,^26^,^27^,^28^. These techniques have been used to hierarchically organize nanoporous materials for sensor arrays, micro fluidic or photonic systems. But they have not been studied for patterning energy storage materials such as nanoporous carbons, and may have shortcomings for the integration of such materials into micro systems. Indirect approaches such as soft lithography^21^ suffer from complex process involving additional moulds that have to be patterned in advance. Some approaches including pen lithography, ink-jet printing and electron beam lithography write or print the patterns of functional materials directly^22^,^23^,^24^,^25^,^26^,^27^,^28^. But they are not efficient approaches that are designed for wafer-level processing, which is the key to batch fabrication of micro devices. In addition, the formed nanoporous oxides have limited thicknesses usually less than 1 μm, and the patterns have limited resolutions usually about hundreds of micrometers.

Here we incorporate nano templating method into microelectronics technology and present the direct prototyping of nanoporous carbon membranes using photopatternable nanocomposite, so that such functional materials can be patterned at wafer level and efficiently integrated into micro devices. Meanwhile, nanoporous polymer can also be produced from the precursor of the carbon for other applications. The novel method has the following features: (1) Three-dimensional (3D) nanoporous membranes with significant thicknesses can be rapidly prototyped, so that such functional materials can provide high surface areas in limited footprint area. (2) The whole process is at wafer level, which is the key to batch fabrication of functional materials on chips. (3) The material can be integrated with existing micro systems, because it can be patterned into arrays, electronically connected with other parts, and the fabrication is based on readily available microfabrication equipments. As a further demonstration, we also specify the direct application of highly nanoporous carbon membranes in micro supercapacitors for energy storage.

The general template synthetic procedure for porous polymers or carbons has the following steps^17^: 1) preparation of the carbon precursor/inorganic template composite, 2) cross-linking for polymers or cross-linking and then carbonization for carbons, and 3) removal of the inorganic template. The space once occupied by the template becomes pores in the resulting material, and the pore structure is the replication of nano structure of the inorganic template. The basic idea in this work, which enables the direct photolithography of the porous materials, is illustrated in Fig. 1. We assume that if a photosensitive polymer was used as precursor for porous polymer or carbon, the composite of the polymer and nano template could not only retain the photosensitibity, but also become nanoporous through general template synthetic procedure.

To demonstrate the idea, we used epoxy-based SU-8^29^,^30^, which is a commonly used negative photoresist, as the photosensitive polymer illustrated in Fig. 1, and nano silica spheres with a uniform diameter of 30 nm as the template (Supplementary Methods and Fig. S1). SU-8 photoresist consists of EPON® Resin SU-8 as basic material, a triaryl sulfonium salt as photoinitiator, and an organic solvent (Supplementary Methods). The epoxy has an initial molecular weight of around 7000, and when exposed to ultraviolet light, it will form a highly structured cross-linked matrix. The high sensitivity, high resolution, low optical absorption, good thermal stability and chemical resistance make SU-8 a widely used material for microfabrication of high aspect-ratio structures, micro-structured molding, packaging and so on^29^,^30^. Moreover, SU-8 can retain micro patterns after high-temperature carbonization, which has been systematically studied by Madou's group and used in the technology of carbon micro electromechanical systems (CMEMS)^31^,^32^,^33^.

## Results

### Patterning and observation of the nanoporous membranes

**Figure 2:**
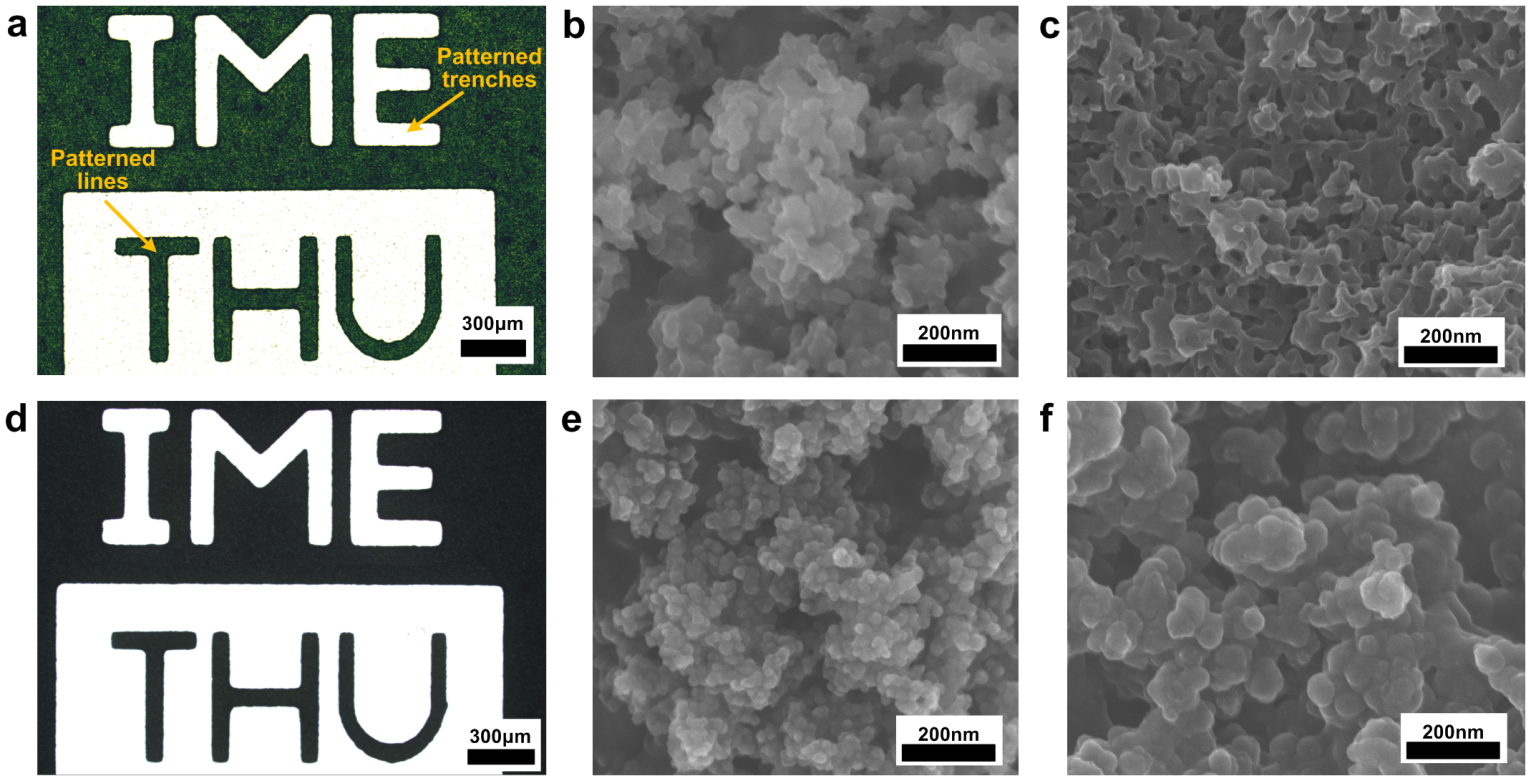
Photographs and SEM images of the patterned membranes. (a) Photograph of the patterned SU-8/silica composite membrane, indicating that the composite can be photo patterned almost as good as regular photoresist. (b) SEM image of the micro structure of the SU-8/silica composite. (c) SEM image of the nanoporous structure of SU-8 after silica is removed from the former SU-8/silica composite. (d) Photograph of the patterned carbon/silica composite membrane, which is obtained by pyrolysis of the patterned SU-8/silica composite at 900°C in nitrogen. (e) SEM image of the micro structure of the carbon/silica composite. (f) SEM image of the nanoporous structure of carbon after silica is removed from the former carbon/silica composite.

In the present work, the silica-templated SU-8 (denoted as STS-x, x is the weight proportion of silica in the silica/SU-8 composite, regardless of the solvent) is formed by uniformly dispersing nano silica spheres in the photoresist, after which the composite is photopatterned as regular photoresist (Supplementary Methods). The adding of silica changes properties of original photoresist, resulting in increase of viscosity and decrease of transparency. Viscosity can be adjusted by varying the content of solvent of the composite and the problem of lower transparency can be overcome by applying higher exposure dose. Fig. 2a shows the photo of STS-0.2 membrane after photolighography, in which the micrometer-sized patterns consisting of trenches and lines are successfully shaped. The nanostructured surface of the membrane is observed as in scanning electron microscopic (SEM) image in Fig. 2b. Further increase of silica weight proportion still maintains the feasibility of photolithography, but the process is accordingly harder to control (Supplementary Fig. S2 and Fig. S3). A disordered and interconnected nanoporous structure inside the polymer membrane, as shown in Fig. 2c, is eventually obtained after etching of the silica template.

To synthesize carbon in situ, the patterned SU-8/silica composite is pyrolyzed at 900°C in nitrogen and becomes carbon/silica composite which keeps the patterns as in Fig. 2d and has the nanostructure shown in Fig. 2e. The porous carbon is then obtained by etching the silica, and nanoporous structure is observed in Fig. 2f. The carbonization of SU-8 and the removal of the silica are evident from thermogravimetric analysis (TGA) and energy dispersive spectroscopy (EDS) tests, and Raman spectroscopy indicates that the synthesized carbon is amorphous (Supplementary Fig. S4 and Fig. S5).

**Figure 3:**
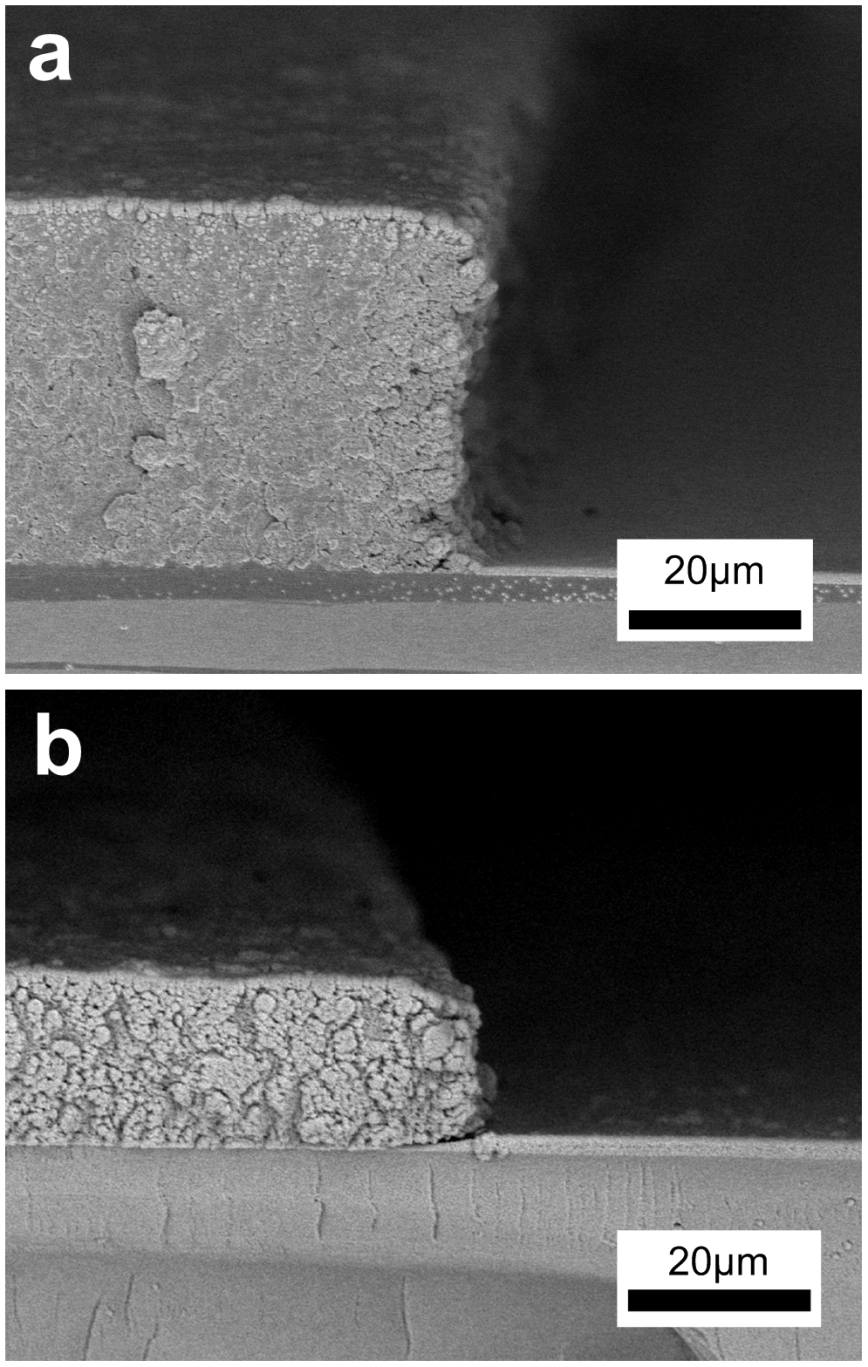
SEM images of the cross-section of the patterned membranes. (a) The edge of a 40 μm-thick SU-8/silica membrane. (b) The edge of a 16 μm-thick carbon/silica membrane carbonized from (a). Vertical shrinkage of the membrane is caused by pyrolysis of SU-8.

The cross-sections of patterned membranes are shown in Fig. 3, in which the SU-8/silica membrane has a considerable thickness of 40 μm, and it shrinks to about 16 μm after carbonization. The etching of silica causes no detectable thinning of the membrane, which means the derived porous membranes are as thick as the composite membranes. In fact, SU-8 photoresist can achieve membrane thicknesses of more than 200 μm by a single spin-coating process and build three-dimensional structures with high aspect ratios of more than 10:1 through lithography^30^. The SU-8/silica composite is also available for thick membranes because the coating process is the same as ordinary SU-8. The aspect ratios of structures fabricated by the composite, however, can not reach those built by pure SU-8, because the nano silica spheres dispersed in the composite cause scattering of lights similar to Tyndall effect happens in colloid during exposure process. Therefore, the actual aspect ratio that can be achieved is related to the content of silica in SU-8/silica composite. Based on our experiments, an aspect ratio of 1:1 for porous SU-8 membranes no thicker than 100 μm is achievable by using STS-0.2. The aspect ratio of porous carbon membranes is accordingly about 0.4:1.

### Surfaces areas and pore size distributions of the nanoporous materials

**Figure 4:**
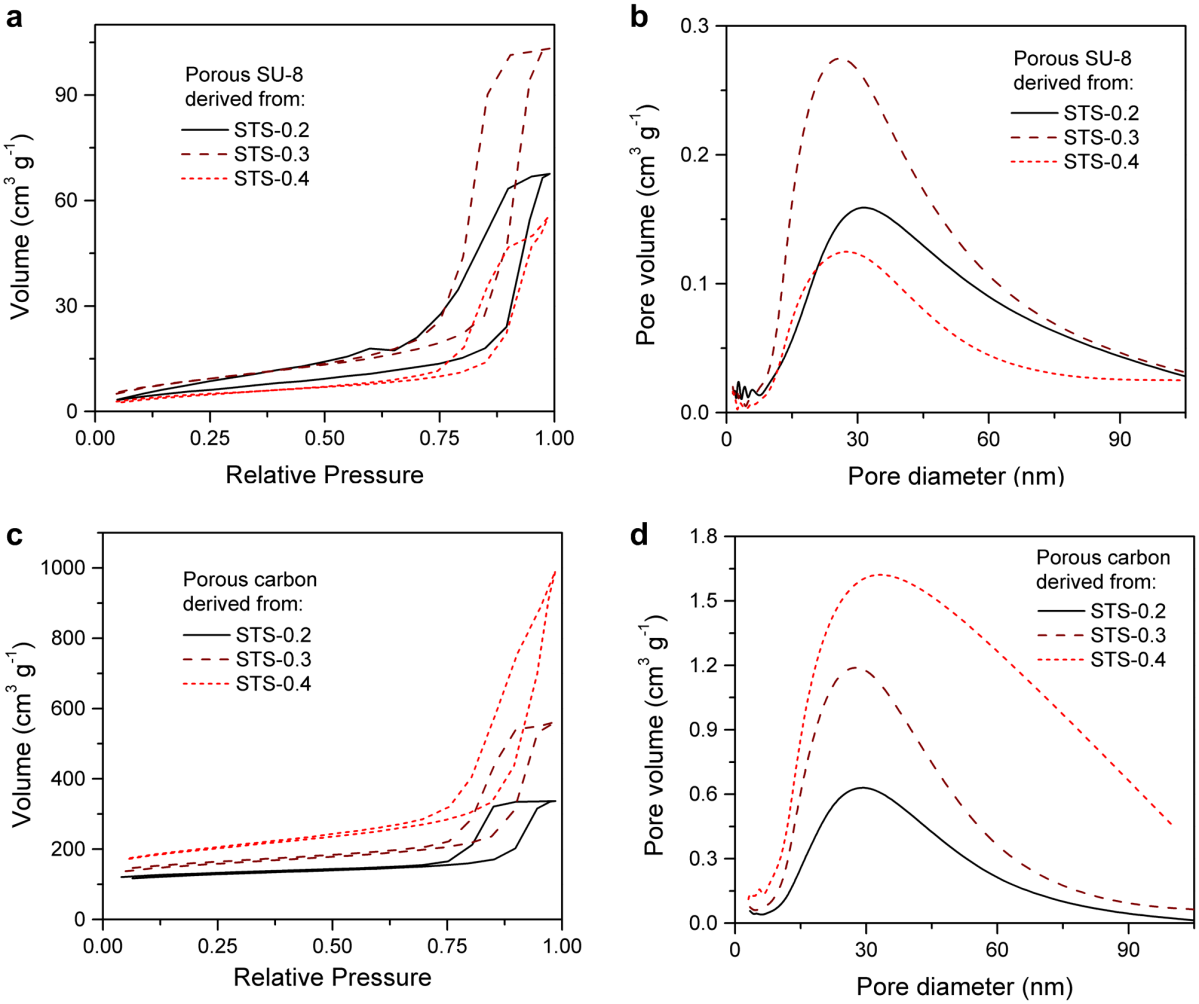
Materials characterization by nitrogen physisorption. Nitrogen adsorption–desorption isotherms of porous SU-8 (a) and porous carbon (c) derived from different precursors. Pore size distributions of porous SU-8 (b) and porous carbon (d) derived from different precursors, calculated based on the BJH method using the adsorption isotherm.

The porosity of the nanoporous material can be tuned by changing the template or simply adjusting the content of each constituent. Three samples, in which the contents of silica in the initial SU-8/silica composites are 20wt% (STS-0.2), 30wt% (STS-0.3) and 40wt% (STS-0.4), respectively, were prepared as precursors for porous polymer or carbon membranes. The specific surface areas and pore structures of porous materials derived from different precursors were characterized by nitrogen physisorption and are summarized in Fig. 4. They have similar pore size distributions, in which there is a peak around 30 nm corresponding to the diameter of nano silica spheres. The distributions indicate that most of the pores are mesopores with pore sizes less than 50 nm, and some macropores larger than 50 nm are generated because of aggregation of nano template spheres. Narrower pore size distributions could be achieved by dispersing nano silica more uniformly. Surface modifications of silica involving surfactants that can react with epoxy groups in SU-8 molecules were not used because it would reduce the feasibility of photolithography of the composite. One solution may be applying stronger dispersion methods than stirring and ultrasonic agitation we have applied. Another phenomenon is that the porosity of both porous polymer and carbon is increasing with the increase of content of silica in the precursor. One exception is porous SU-8 derived from STS-0.4, which has the lowest porosity. It can be explained by the collapse of walls between pores, which are too thin to support the porous structure replicated from silica. Unlike the polymers, the carbons are hard enough to support the porous structure and have considerable surface areas (Supplementary Table S1) ranging from 393 m^2^ g^−1^ (derived from STS-0.2) to 634 m^2^ g^−1^ (derived from STS-0.4).

The volumetric surface area is of more significance for applications in micro systems where the space is limited. We estimated the mass densities of the porous carbon membranes here, which turn out to be 0.74 g cm^−3^ (derived from STS-0.2) and 0.48 g cm^−3^ (derived from STS-0.4), and then calculated the volumetric surface areas, which are 291 m^2^ cm^−3^ (derived from STS-0.2) and 304 m^2^ cm^−3^ (derived from STS-0.4) respectively.

It has been reported that the gravimetric surface area of porous carbon can reach a value as high as 3100 m^2^ g^−1^ for activated graphene^34^. However, the activated graphene exhibits a low mass density of ~0.1 g cm^−3^ as powders, which leads to a volumetric surface area of 310 m^2^ cm^−3^, the same level compared to our nanoporous carbon membranes. In fact, the monolithic nanoporous carbons here do not have interparticle porosity and therefore shows volumetric surface area comparable to powders with extremely high gravimetric capacitances. Also, the nanoporous carbon membranes can be applied directly without additional polymer binders and high pressure process for the assembly of powders. The polymer binders will block part of the nanoporous structure and further reduce the applicable surface area of powders, and the high pressure process is not valid for the fabrication of micrometer-sized electrodes by using existing technologies.

### Application of nanoporous carbon membranes for micro supercapacitors

**Figure 5:**
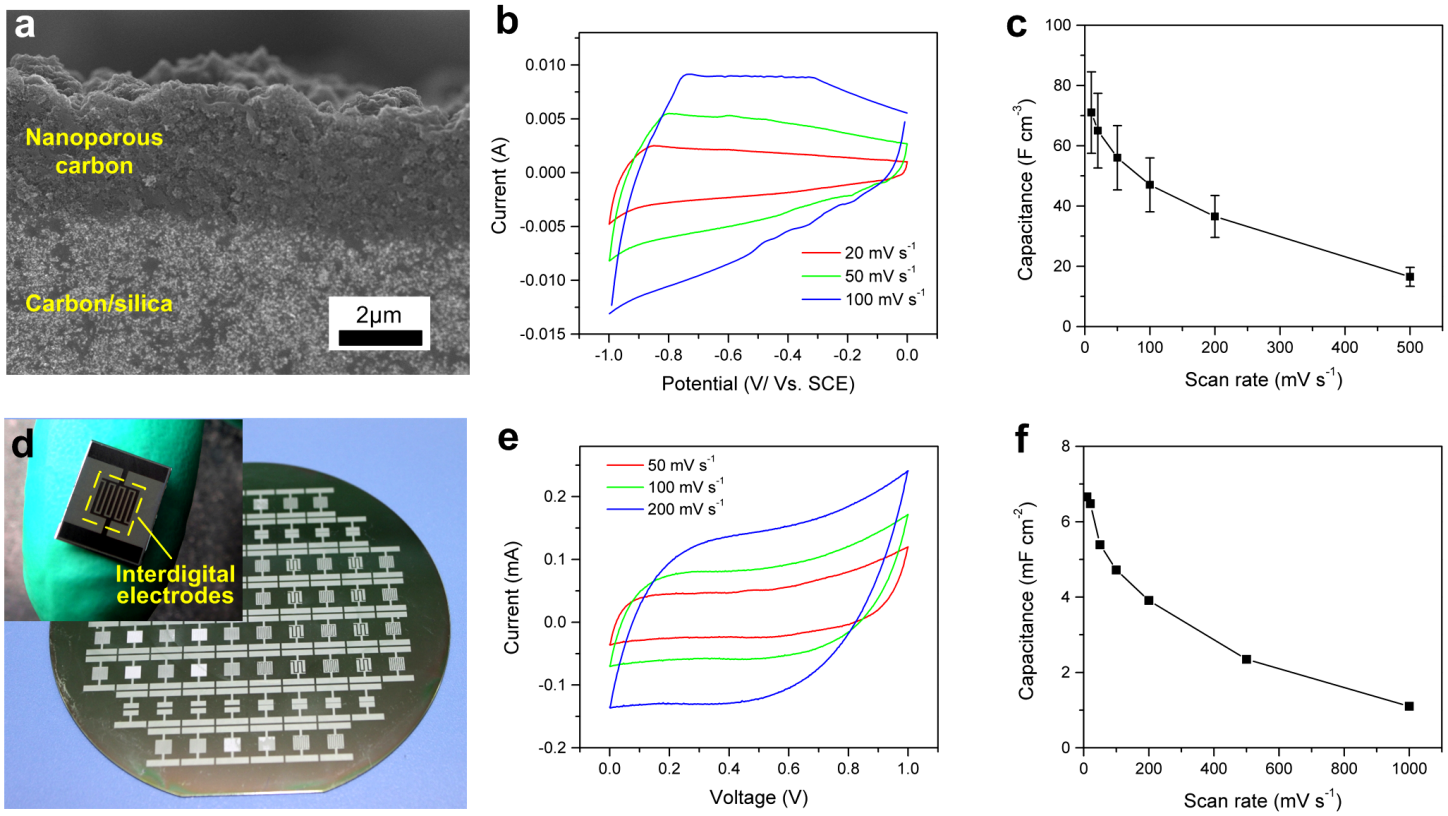
Nanoporous carbon membranes as electrodes for supercapacitors. (a) SEM image of the cross-section of a half-etched carbon/silica membrane, in which the silica is uniformly etched from the top of the membrane. (b) Cyclic voltametry curves of a 20 μm-thick membrane in a three-electrode system using 0.2 M K_2_SO_4_ as electrolyte. (c) Calculated volumetric capacitances of monolithic nanoporous carbon membranes. (d) A wafer with different electrode patterns after one-step lithography of silica-templated SU-8. The inset photograph shows a micro supercapacitor prototype fabricated using the same process as a nanoporous carbon membrane. The device has 8 interdigital fingers as electrodes, each finger is 320 μm wide and separated by 160 μm-wide space. (e) Cyclic voltametry curves of a micro supercapacitor using 0.2 M K_2_SO_4_ as electrolyte. (f) Calculated specific capacitances of the micro supercapacitor from cyclic voltametry curves.

The obtained nanoporous materials can be used for multiple applications. Here we demonstrate the direct use of the nanoporous carbon membrane as electrodes for micro electrical double-layer (EDL) supercapacitors, which are expected to address the need for microscale energy storage on chips^11^,^35^,^36^,^37^,^38^,^39^. As the etching of carbon/silica membrane for nanoporous carbon can be well controlled as other wet etching processes for microfabrication (Fig. 5a), we fabricated nanoporous membranes with different thicknesses ranging from 3 μm to 20 μm and tested their electrochemical behavior in a three-electrode system. Fig. 5b shows the cyclic voltametry curves of a 20 μm-thick membrane, indicating a capacitive behavior under various scan rates. Calculations verify that the capacitance is proportional to the volume of the nanoporous layer, independent of the thickness. The calculated volumetric capacitances of membranes with different thicknesses are plotted as a function of scan rate in Fig. 5c.

As the precursor of nanoporous carbon membrane can be directly patterned at wafer level, as presented in Fig. 5d, micro supercapacitors with designed electrode configurations can be rapidly prototyped and batch fabricated. A micro supercapacitor with 5 μm-thick active electrodes before package is shown in the inset photo of Fig. 5d. The capacitive behavior of the prototype is indicated by cyclic voltamety curves shown in Fig. 5e and the calculated capacitances are plotted in Fig. 5f. The prototype has a specific capacitance of about 6.7 mF cm^−2^, or a volumetric capacitance of 13.4 F cm^−3^ considering the active thickness of the electrodes, at the scan rate of 10 mV s^−1^ (see Supplementary Methods for details). The capacity of our prototype, in terms of both capacitance per area and capacitance per volume, is larger than those of micro supercapacitors fabricated using state-of-the-art technologies such as laser writing (0.51 mF cm^−2^, 3.1 F cm^−3^)^35^, electrophoretic deposition (1.7 mF cm^−2^, 1.3 F cm^−3^)^36^ and printing (2.1 mF cm^−2^, 2.7 F cm^−3^)^37^, and it proves that our novel method has great potential in batch fabricating high-performance micro energy storage electrodes on a chip. The micro supercapacitors developed here can also be electronically connected and easily integrated with other micro systems. A little design on the fabrication process can improve the electronic connection (Supplementary Fig. S6 and Fig. S7).

## Discussion

The monolithic polymer membranes with disordered and interconnected porous structure are chemically and mechanically stable when SU-8 molecules are totally cross-linked^30^. They can be used in micro fluidic systems to trap or filter biomolecules, and in micro sensors to provide high-surface-area support for enzymes or catalysts. The porous carbon membranes, on the other hand, provide conductive and supportive nanostructured frames that allow for even more applications after decorations^40^,^41^.

In the application for electrodes of EDL supercapacitors, the monolithic carbon membranes with controlled nanoporous structure not only show merits over powder-like materials as described in the Results section, but also are better than micro carbon pillars directly derived from photoresist^31^,^39^, because they offer much larger surface areas inside the nanoporous structure. Although Beidaghi and coworkers^39^ show that micro carbon arrays have considerably larger capacitance after electrochemical activation, the nanoporous carbon membranes described here still have larger volumetric capacitances and avoid the detrimental activation process. Other continuous porous carbon membranes based on carbide derived carbon (CDC) materials have also been demonstrated to be attractive for micro supercapacitors^11^. However, they suffer from prototyping problem, i.e. patterning of three-dimensional micro electrode structures on CDC is a challenge. Our prototypes can not only be directly patterned, but also avoid time-consuming chemical vapor deposition (CVD) for thick carbide membranes and high-cost dry-etching process for CDC^11^. We have also shown in the Results section that our micro supercapacitor has superior performance over similar devices fabricated using state-of-the-art technologies. And moreover, the performance of our prototype has great potential for improvement. As the capacitance per footprint area is proportional to the thickness of electrodes, and the SU-8 photoresist can achieve considerable membrane thicknesses, the capacity of our prototype can be directly improved by fabrication of thicker electrodes. While the nanoporous carbon membranes alone provide high EDL capacitance, they can also be used as porous supporters for other energy storage materials. For example, deposition of nano-sized metal oxide particles onto the inside surface of the membranes can greatly increase their volumetric capacitances.

In conclusion, we have successfully demonstrated the use of photopatternable silica-templated SU-8 for direct prototyping of nanoporous polymer or carbon at wafer level, and specified the direct application of the nanoporous carbon as electrodes for on-chip energy storage devices. The micro supercapacitor prototype shows high capacity and great potential for improvement. The idea can be extended to other photosensitive polymer/inorganic template nanocomposite systems to derive a new series of nanoporous materials. By changing the template material, pores with different sizes and even ordered porous structures can be generated inside the photopatterned polymer or carbon. Improvements in scalability, compatibility and cost make it a promising technology for various applications, especially energy storage, in integrated micro systems.

## Methods

### Preparation of the materials

The typical preparation procedure of the STS-0.2 composite is detailed as below. 7 g of SU-8 2010 (MicroChem Corp.), which contains about 4 g of SU-8 epoxy resin, was directly mixed with 1 g silica under magnetic stirring. 2 g of cyclopentanone was added as solvent to adjust the viscosity. The mixture was stirred for more than 2 h, under ultrasonic agitation for 1 h, and kept standing for more than 2 h before use. Preparation of other composites, such as STS-0.3 or STS-0.4, was similar but different only in the weight proportion of silica. The mixture was then spin-coated on a silicon wafer at a spin speed of around 1500 r/min. The wafer was baked at 95°C for 3 min to evaporate part of the solvent, and then exposed to ultraviolet light covered with a photomask. Another bake at 95°C for 3 min was applied before development, and the bake at 150°C for 3 min was applied to further cure the material after development. Porous polymer membrane was derived by etching the silica using the solution that consists of 4wt% HF, 46wt% deionized water and 50wt% ethanol. Porous carbon membrane, on the other hand, was obtained by first carbonizing the membrane at 900°C in nitrogen for 1 h, and then etching the silica using the same solution.

### Characterization of the materials

The on-chip membrane during each step was observed by a photo microscope (Olympus MX51) and an ultra-high resolution scanning electron microscope (Hitachi S-5500) equipped with an X-ray energy-dispersive spectroscopy (EDS). Raman spectroscopy was conducted by a microscopic confocal Raman spectrometer (Renishaw, RM2000). Samples for TGA and surface area analysis were prepared by peeling off the composite before the curing step. The porous polymer and carbon samples are derived using the same process as that of the on-chip membranes. TGA was conducted by heating samples from room temperature to 900°C at a rate of 10°C/min in nitrogen, and then keeping the samples at 900°C in oxygen, using a thermal analyzer (NETZSCH STA 409). Surface area and pore structure of the samples were analyzed by nitrogen physisorption (QuadraSorb SI).

### Electrochemical characterization of nanoporous carbon membranes and the micro supercapacitor prototype

Cyclic voltametry (CV) tests of the on-chip membranes were conducted in a three-electrode electrochemical system with the as-synthesized porous carbon membrane on the silicon substrate as the working electrode, a Pt foil as the counter electrode, saturated calomel electrode as the reference electrode, and 0.2 M K_2_SO_4_ solution as the electrolyte. CV curves of the as-fabricated micro supercapacitor were measured after the prototype was sealed by a PDMS cap with 0.2 M K_2_SO_4_ injected into the interdigital electrodes area. Data were recorded by an electrochemical workstation (CHI 860D).

## Author Contributions

C.S. and X.W. conceived the idea of incorporating nano templating method into photolithography technology. C.S., W.Z. and F.K. designed the detailed preparation process and characterization methods for the materials. C.S. and X.W. designed the prototype of micro supercapacitor and related measurements. C.S. and W.Z. conducted the fabrication and experiments of the materials and the devices. C.S., X.W., W.Z. and F.K. co-wrote the paper, and discussed the results and commented on the manuscript.

## Supplementary Material

Supplementary Information Supplementary Information

## Figures and Tables

**Figure 1:**
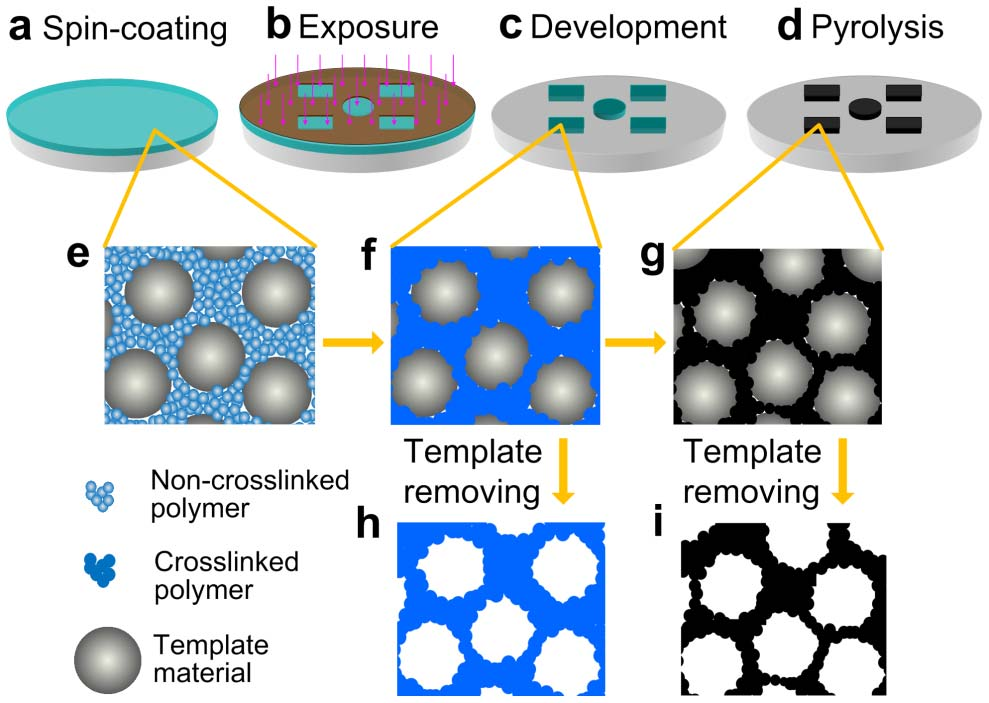
Schematic diagram of the photopatterned nanoporous materials. (a) Nanocomposite of photosensitive polymer and template material is first spin coated on a substrate. (b) The composite membrane is exposed to light through a photomask. (c) The patterns on the photomask is transferred to the membrane, because the polymer being exposed to light becomes crosslinked and stays on the substrate, and the unexposed part is removed after development. (d) Patterned carbon membrane is formed after pyrolysis of the composite membrane under high temperature in inert atmosphere. (e–g) Illustration of the nanostructure of the composite in each step. Porous polymer (h) or porous carbon (i) can be obtained by removing the template after the corresponding steps.
