# Supplementary material for: Direct Prototyping of Patterned Nanoporous Carbon: A Route from Materials to On-chip Devices
Source: Sci Rep. 2013 Jul 26;3:2294. doi: 10.1038/srep02294 (PMC3724177; doi:10.1038/srep02294)
Supplement: Supplementary Information [file srep02294-s1.pdf]

Supplementary Information

Direct Prototyping of Patterned Nanoporous Carbon: A Route from Materials to On-chip Devices

Caiwei Shen[1,2], Xiaohong Wang[1,2], Wenfeng Zhang[1,3] and Feiyu Kang[1,3]*

[1]Tsinghua National Laboratory for Information Science and Technology, PR China

[2]Institute of Microelectronics, Tsinghua University, Beijing 100084, PR China

[3]Department of Materials Science and Engineering, Tsinghua University, Beijing 100084, PR China

SUPPLEMENTARY METHODS

Additional Information of SU-8 and silica used in the present work

The SU-8 photoresist used in this work is chosen from SU-8 2000 series sold by MicroChem Corp.. The SU-8 2000 series consist of EPON® Resin SU-8 (Shell Chemical) as the main material, triaryl sulfonium salt as the photoinitiator, and cyclopentanone as the solvent. The weight proportion of each composition is estimated from data in Material Safety Data Sheets of the products

The nano silica is bought from Beijing DK Nano, Ltd. and directly used to mix with the photoresist. The silica is naturally epoxy-philic without any treatment by surfactants. The silica is about 30nm in diameter, as shown in TEM image in Figure S1. Its Fourier transform infrared (FTIR) spectroscopy is measured and the result is shown in Figure S1.

Silica has been applied to modify the SU-8 photoresist in previous studies.[1-3] It has been proved that some of the properties of SU-8, such as sensitivity, adhesion and mechanical stability, can be improved by adding a small amount of silica (less than 2.5wt%). In this work, however, larger proportion of silica (more than 10wt%, as far as we know) has to be added to generate interconnected nanoporous structure, at the sacrifice of resolution of photolithography.

Specific capacitance calculation

The capacitances (in F) of either the nanoporous carbon membranes or the micro supercapacitor prototypes were calculated using the measured cyclic voltammetric curves over the whole potential range according to the following equation:

$$C_o = \frac{\int Idt}{\Delta V} = \frac{\int IdV}{\Delta V \times r} \qquad (1)$$

where *I* is the recorded current, *t* is the time, *V* is the potential or voltage, and *r* is the scan rate.

The volumetric capacitance of the carbon membrane (in F cm^{-3}) was calculated by dividing C_o by the volume of nanoporous layer, and the specific capacitance of the prototype (in mF cm^{-2}) was calculated by taking into account only the projected surface area of the interdigital electrodes and the space between them (3.68 mm × 3.56 mm).

SUPPLEMENTARY FIGURES

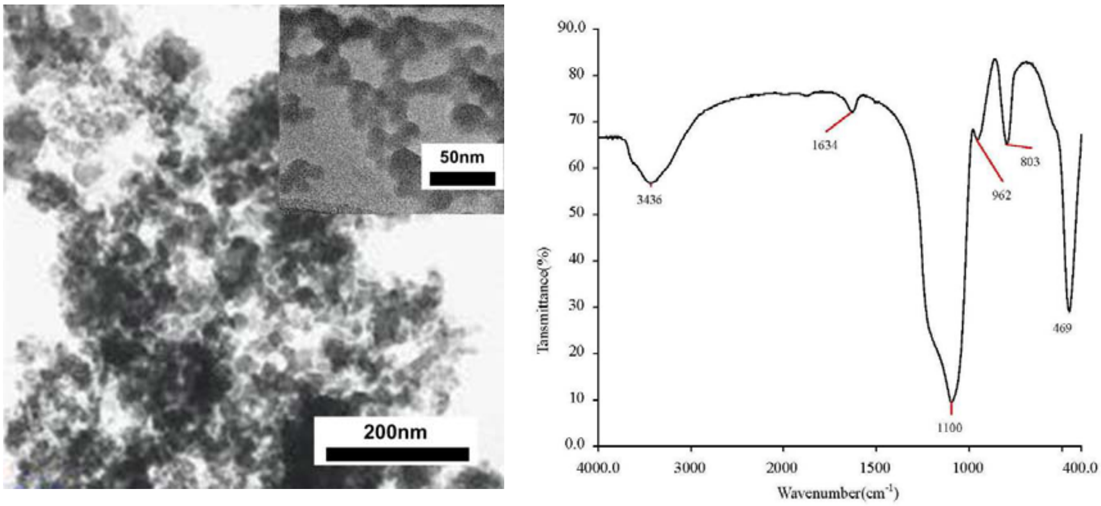

Supplementary Figure S1. TEM image (left) and Fourier transform infrared (FTIR) spectrum (right) of the nano silica sample.

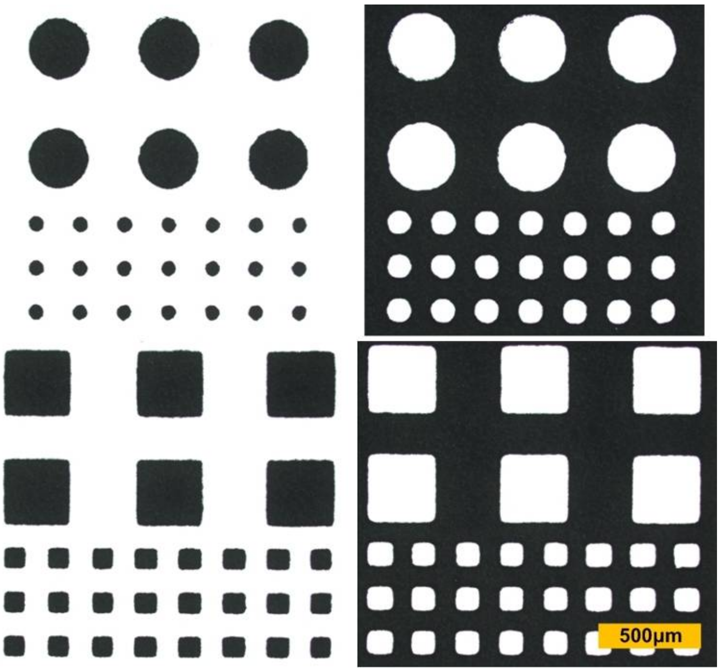

Supplementary Figure S2. Porous carbon membrane derived from STS-0.2 with array patterns.

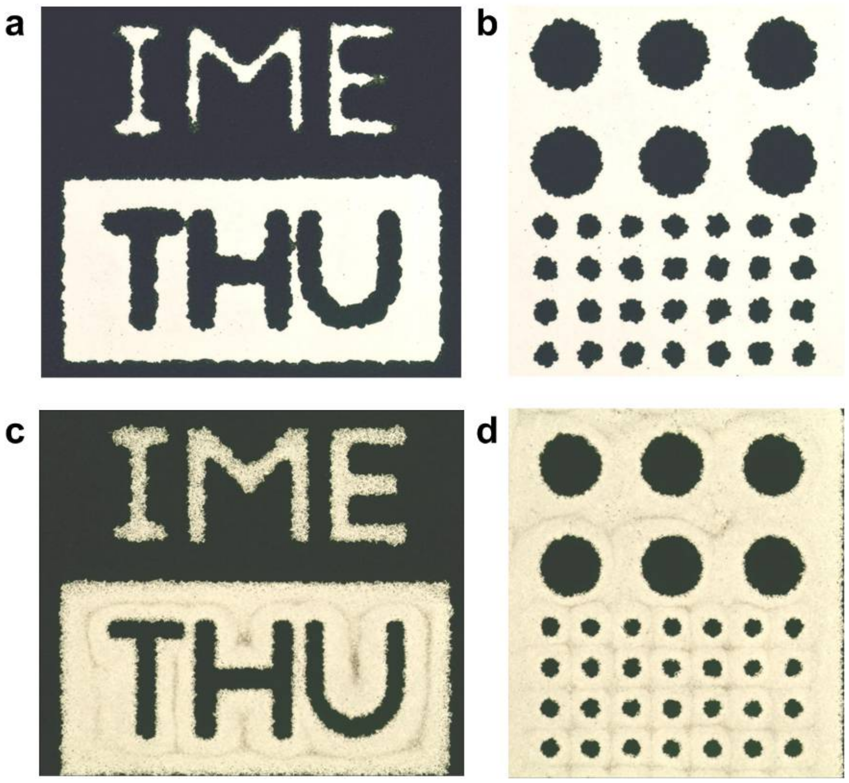

Supplementary Figure S3. Patterned porous SU-8 membranes derived from STS-0.3 (**a, b**) and STS-0.4 (**c, d**) without optimization of the lithography process. They are all exposed to ultraviolet with the same dose of 1080 mJ cm^{-2}. It appears that the membrane derived from STS-0.3 is over exposed, which causes the expansion of lines and the contraction of trenches. The problem of the membrane derived from STS-0.4, however, lies in the difficulty of removing the unexposed part. This is due to the scattering of light by nano silica during exposure. The polymer membranes appear to be black under the photo microscope because their surfaces are rough and do not reflect light.

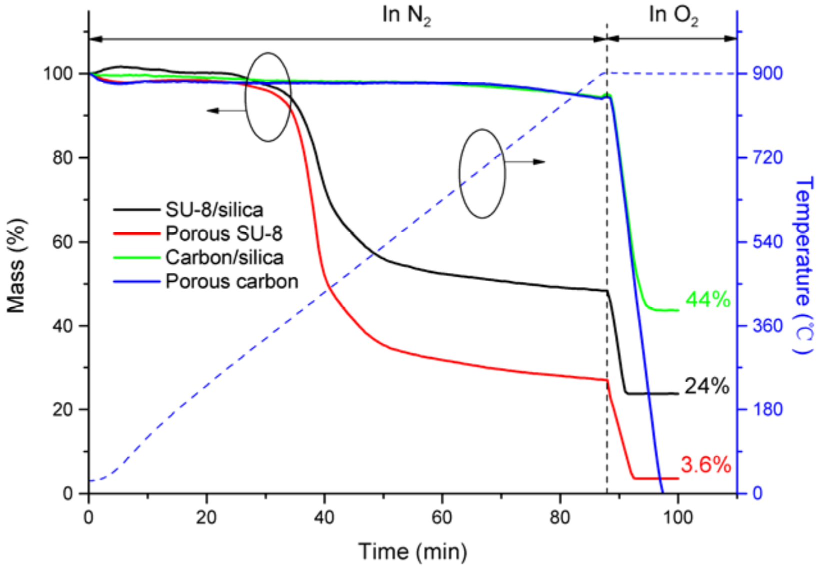

Supplementary Figure S4. Materials characterization by TGA. Four samples derived from the same precursor, STS-0.2, are first heated in N_2 from room temperature to 900 °C, and then burned in O_2 at 900 °C. The mass loss of SU-8/silica composite and porous SU-8 in N_2 is caused by decomposition of SU-8. SU-8 begins to pyrolyze at about 300℃, becomes carbonized around 600℃ and loses not much weight from 600℃ to 900℃ in nitrogen. It can be calculated that nearly 70wt% of SU-8 is lost from room temperature to 900℃, independent of the content of silica. The weight losses of all samples in oxygen are caused by combustion of carbon and organic matters, and the final weight is the content of silica. The porous SU-8 sample has 3.6wt% retention at 900℃ in oxygen, probably because part of the silica is surrounded by the polymer and not totally removed by etching. The total weight loss of the porous carbon, on the other hand, indicates the removal of all the silica.

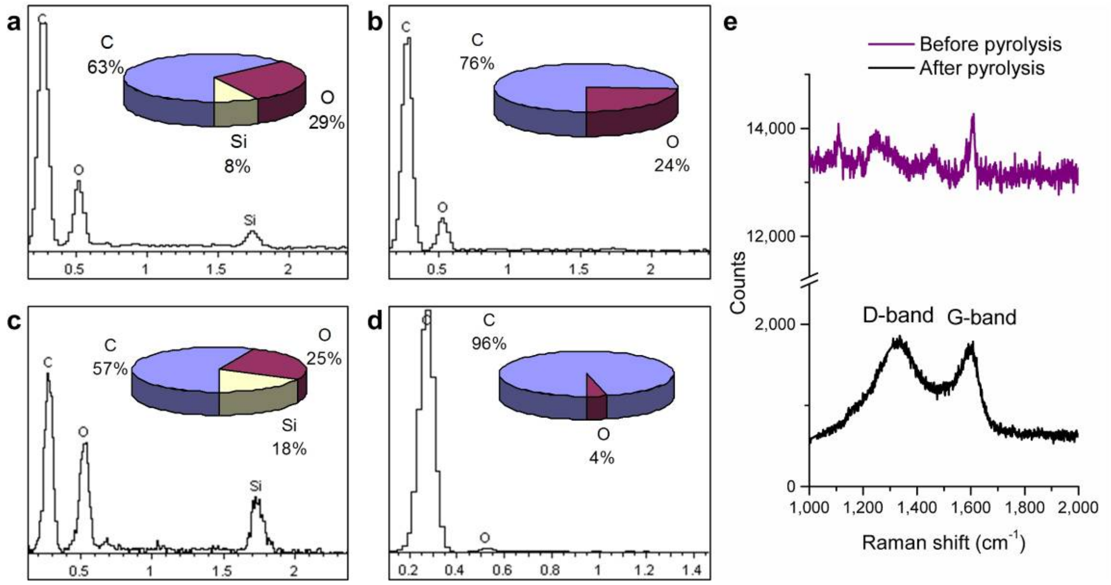

Supplementary Figure S5. a-d, EDS analyses of different materials (**a**): SU-8/silica composite (**b**): porous SU-8 (**c**): carbon/silica composite (**d**): porous carbon. The unit of the lateral axis is keV. **e,** Raman spectra of the SU-8/silica composite before and after pyrolysis. The unpyrolyzed composite is strongly fluorescent, and an amorphous carbon structure is observed after pyrolysis.

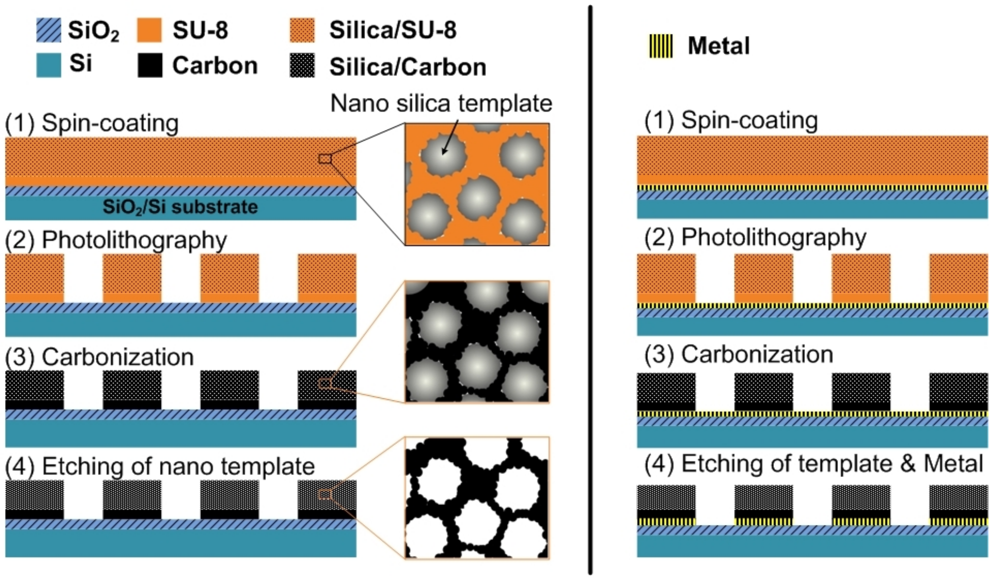

Supplementary Figure S6. Fabrication processes of micro supercapacitors on SiO_2/Si substrate (left figure) or metal/SiO_2/Si substrate (right figure), where the metal layer can be high-melting-point metal such as Mo. A layer of pure SU-8 is coated at the bottom at first and is finally carbonized to be a carbon layer, which acts as current collector as well as insulation layer during etching process.

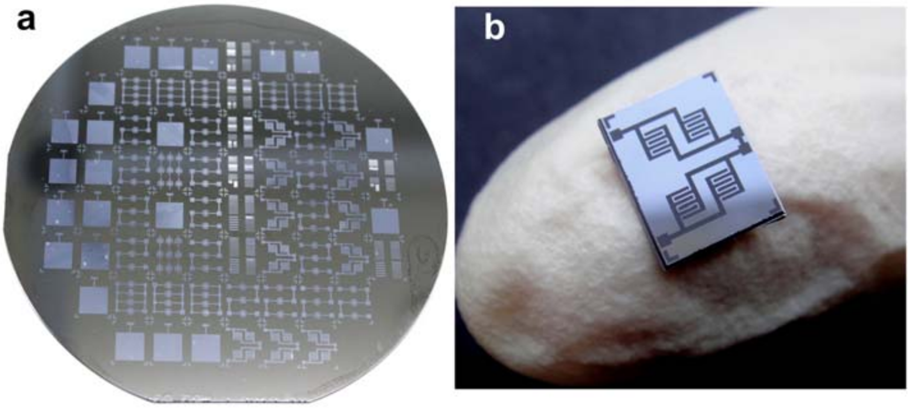

Supplementary Figure S7. (**a**) Photo of a wafer with different electrode patterns, including micro supercapacitor arrays, after one-step lithography of silica-templated SU-8. (**b**) A cell with four micro supercapacitors connected together. Every two micro supercapacitors are first connected in parallel and then in series.

SUPPLEMENTARY TABLES

Material	Porous SU-8			Porous Carbon		
Silica content in precursor	20%	30%	40%	20%	30%	40%
BET surface area (m^2g^{-1})	22	33	18	393	492	634
Pore Volume (cm^3g^{-1})	0.11	0.16	0.09	0.53	0.87	1.53

Supplementary Table S1 Gravimetric surface area information calculated from nitrogen adsorption–desorption isotherms.

Supplementary References

(1) Jiguet, S., Bertsch, A., Judelewicz, M., Hofmann, H. & Renaud, P. SU-8 nanocomposite photoresist with low stress properties for microfabrication applications. Microelectronic Engineering 83, 1966-1970 (2006).

(2) Chiamori, H. C., et al. Suspension of nanoparticles in SU-8: Processing and characterization of nanocomposite polymers. Microelectron.J. 39, 228-236 (2008).

(3) Suter, M., et al. A photopatternable superparamagnetic nanocomposite: Material characterization and fabrication of microstructures. Sensors and Actuators B-Chemical 156, 433-443 (2011).
